# Coupling Musculoskeletal Dynamics and Subject-Specific Finite Element Analysis of Femoral Cortical Bone Failure after Endoprosthetic Knee Replacement

**DOI:** 10.1155/2019/4650405

**Published:** 2019-02-20

**Authors:** Fuhao Mo, Haotian Zhang, Siqi Zhao, Zhi Xiao, Tang Liu

**Affiliations:** ^1^State Key Laboratory of Advanced Design and Manufacture for Vehicle Body, Hunan University, Changsha, Hunan 410082, China; ^2^Department of Orthopedics, The Second Xiangya Hospital of Central South University, 139 Renmin Road, Changsha, Hunan 410011, China

## Abstract

**Background and Objective:**

A common reconstruction procedure after a wide resection of bone tumors around the knee is endoprosthetic knee replacement. The aim of this study was to investigate the characteristics of bone injury of the patient after endoprosthetic knee replacement during walking.

**Methods:**

A subject-specific finite element model of the femur-prosthesis-tibia complex was established via CT scans. To obtain its physiologically realistic loading environments, the musculoskeletal inverse dynamic analysis was implemented. The extracted muscle forces and ground forces were then applied to the finite element model to investigate bone stress distribution at various stages of the gait cycle.

**Results:**

The maximum femur stress of each stage varied from 33.14 MPa to 70.61 MPa in the gait cycle. The stress concentration position with a distance of 267.2 mm to the tibial plateau showed a good agreement with the patient injury data.

**Conclusions:**

Overall results indicated the reasonability of the simulation method to determine loading environments and injury characteristics which the patient experienced with knee endoprosthesis during walking.

## 1. Introduction

Osteosarcoma is one of the most common primary bone tumors, which mainly happens to children and adolescents [1]. Current treatments for osteosarcoma include surgical resection and multiagent neoadjuvant chemotherapy. These kinds of treatments have significantly improved the survival rate of patients in 5 years. Large bony defects resulting from surgical resection of malignant bone tumors present a large challenge. A common reconstruction procedure after the wide resection of bone tumors around the knee is endoprosthetic knee replacement. However, there are several complications related to endoprostheses, such as infection, loosening, dislocation, and periprosthetic fractures including the anterior cortical perforation of the femur near the stem tip. When it comes to insertion of the intramedullary nail for proximal femur fractures, patients short in stature and those with excessive bowing of femurs have higher risks of anterior impingement and cortical perforation [2]. This risk may also be applied to patients with endoprosthetic knee replacement after a wide resection of bone tumors.

However, the physical mechanisms of bone injury during gait dysfunction are far from being fully understood. Carty et al. [3] pointed out that the muscles played a bigger role on the movement than bone resection through a retrospective outcome study on 20 limb salvage patients (10♂ and 10♀). Okita et al. [4] compared the gait kinematics, kinetics, and energetics of the patients with healthy subjects. The results indicated that patients tended to compensate for dysfunction of the reconstructed knee with the muscles around the ipsilateral ankle and the contralateral hip, with increasing load on the contralateral limb during walking. Hitherto, the traditional experimental study can only detect the patients' kinematics by volunteer tests due to ethics regulation. Stress and muscle force detection are necessary to deeply investigate injury of local penetration.

At present, the three-dimensional finite element analysis method is gradually being widely used in the medical field, involving body injury mechanism analysis, surgical methods, and medical device design. Completo et al. [5] assessed how the femoral stems with different constrained implants can modify the structural behavior of the proximal tibia using finite element models. Baldwin et al. [6] presented a dynamic, force-driven FE knee model, with a full 6-DOF knee joint (both patellofemoral and tibiofemoral joints) including a physiological extensor mechanism and a specimen-specific tibiofemoral ligamentous constraint. Ural et al. [7] developed a new fracture assessment approach combining HR-pQCT imaging with fracture mechanics based on the finite element model to evaluate the distal radius fracture load. Ascenzi et al. [8] presented an innovative method to perform a multiscale finite element analysis of the cortical component of the femur using the individual's computed tomography scan and a bone specimen obtained in conjunction with orthopedic surgery.

Nevertheless, defining the effect of muscle forces in the human gait using an individual finite element model is difficult. Wagner et al. [9] adopted an analytical method combining musculoskeletal dynamics and structural finite element theory to explore the femur response under physiological loads during walking. Although the study was not related to implant consideration or a specific human body model, combining musculoskeletal dynamics and finite element analysis was found to be effective. Thus, the aim of this study was to investigate the characteristics of bone injury of the patient with endoprosthetic knee replacement during walking by combining generic musculoskeletal dynamics and finite element analysis.

## 2. Methods and Materials

### 2.1. Epidemiology and Simulation Method

This study was approved by the Second Xiangya Hospital committee for clinical research (no. 2012-S231), and the informed consent was obtained from the patients and their parents or guardians participating in the study. The patients and their parents or guardians provided written informed consent for the publication of individual clinical details and accompanying images. Between January 2003 and December 2012, anterior cortical perforation of the femur near the stem tip was observed in 9 patients (7 female and 2 male) in the Second Xiangya Hospital. The lesions of these patients were in the distal femur, which were treated by “en bloc” excision and reconstruction of the bone defect with endoprosthetic knee replacement. The result of primary diagnoses of the patients was osteosarcoma. The mean patient age when the femur perforation was first observed was 21.3 years (range of 16-30 years). The average time between surgery and anterior cortical perforation was 6.7 years (range of 3.5-8.9 years).

The coupling musculoskeletal analysis and finite element analysis methods were used to determine the injury mechanism of femoral penetration during a normal gait. The generic musculoskeletal finite element model was obtained based on the anatomy data of the patient. First, the inverse dynamic analysis was implemented to determine the loading environments for the femur-prosthesis-tibia complex based on the musculoskeletal model and experimental data. Then, the finite element analysis with boundary conditions extracted from the inverse dynamic analysis was implemented to analyze the bone stress distribution covering various gait phases.

### 2.2. Establishment of the Subject-Specific Finite Element Model

**Figure 1:**
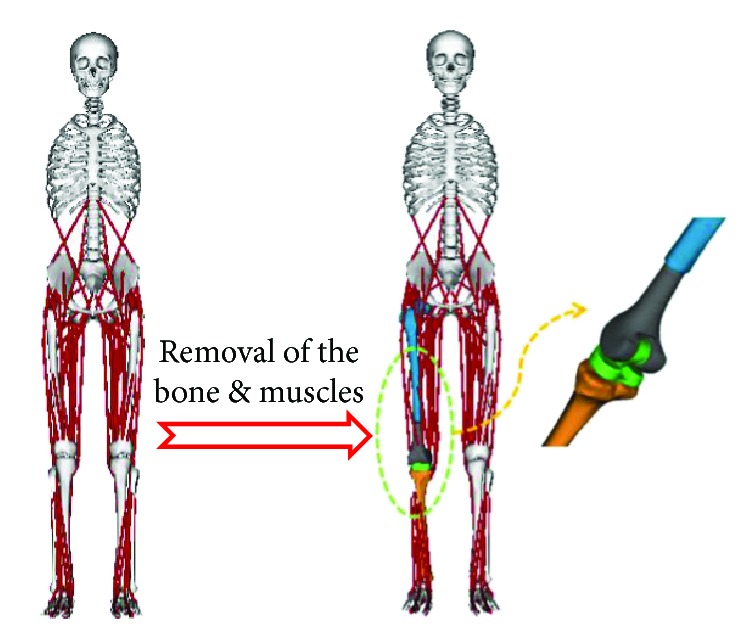
Development of the musculoskeletal model.

**Figure 2:**
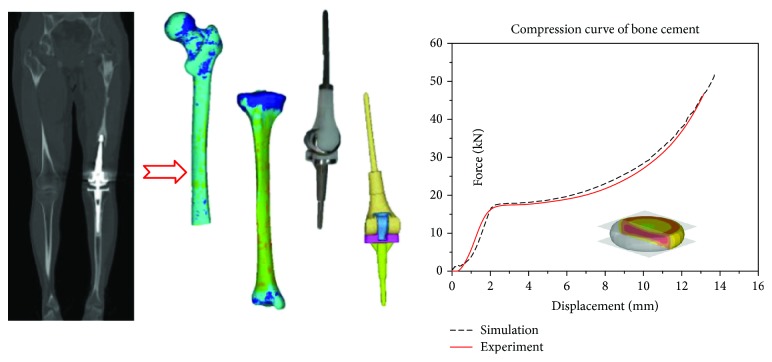
Model geometry reconstruction and finite element modeling.

A subject-specific tibia and femur model was established based on CT scans of an endoprosthetic knee replacement volunteer with femoral penetration (Figure 1) in the Second Xiangya Hospital. The axial interval of the CT scan was 1 mm. An additional 3D laser scan (ATOS Core 200) was implemented for the implant model to determine the detailed geometry of the patient implant. Then, all model geometries were dealt with a CAD modeling package (Geomagic Studio) after 3D digitalization. The geometry of the femur, tibia, and prosthesis was imported into Hypermesh v14.0 (Altair Engineering, USA) to generate a model consisting of tetrahedral elements. The model convergence was tested with mesh sizes from 4 mm to 0.5 mm. To balance the calculation time and stability, an average element edge length of 2 mm was defined for the model meshing (Figure 2).

Based on the gray levels of the CT scan, the 3D mesh models of the femur and tibia were imported into medical modeling software (Mimics 14.0) to define material properties. The bone mechanical properties were determined on the basis of the following equations [10]:

(1) $$\begin{array}{c} D=-13.4+1017\times \text{GV}, \end{array}$$

(2) $$\begin{array}{c} E=-388.8+5925\times D, \end{array}$$

where GV is the gray value of the bone in the CT scan, *D* is the bone density, and *E* is the elastic modulus. The material properties of the prosthesis were obtained from the literature. The compression test of the bone cement was also implemented to determine its properties for finite element simulation. All material properties in the finite element simulation are listed in Table 1.

The finite element model was established based on the LS-DYNA code. Considering the anisotropic and nonlinear properties of the cortical bone, Mat 124 with different tensile and compressive mechanical properties was used to model the cortical bone. The spongious bone was modeled with Mat 105 which consists of an elastic part and an anisotropic viscoplastic part related to continuous damage. The elastic-plastic constitutive model (Mat 24) was selected for the prosthesis. Considering the nonlinear parameters of these material models, we defined yield stress and ultimate strain based on our previous general models [11, 12].

### 2.3. Development and Validation of the Musculoskeletal Model

A three-dimensional musculoskeletal model (3DGaitModel2392) in the OpenSim was used for musculoskeletal analysis. The model consists of 13 segments, 12 links, 23 degrees of freedom mechanical links, and 54 muscle-tendon units which have been validated against volunteer experimental tests [11]. To accurately determine the patient kinematics, the model was adjusted according to the volunteer measurement, detailed surgery process, and CT scan data. The whole model was first scaled to fit the patient size. Then, several muscles were removed according to the surgery such as lateral gastrocnemius, medial gastrocnemius, and vastus intermedius. The femur of the model was cut from the plane which is 3 cm above the tumor boundary, and the tibia was cut from the plane which is 1 cm below the tibial plateau (Figure 1). Then, the prosthesis was implanted and connected with the residual bones.

The musculoskeletal model was evaluated against experimental data from the literature. Okita et al. [4] performed gait analysis for 8 patients who underwent various endoprosthetic knee replacement after bone tumor resection and 8 matched healthy subjects. It presented normalized experimental data in detail regarding the patient size, such as ground reaction force, joint moment, and angle. The results indicated that the peaks of the vertical ground reaction forces (GRFs) and fore-aft GRFs of the patient amputee side were significantly smaller than those of healthy subjects and healthy sides of the patients. The joint angles of the patient amputee side were also evidently different with those in the other two situations.

**Figure 3:**
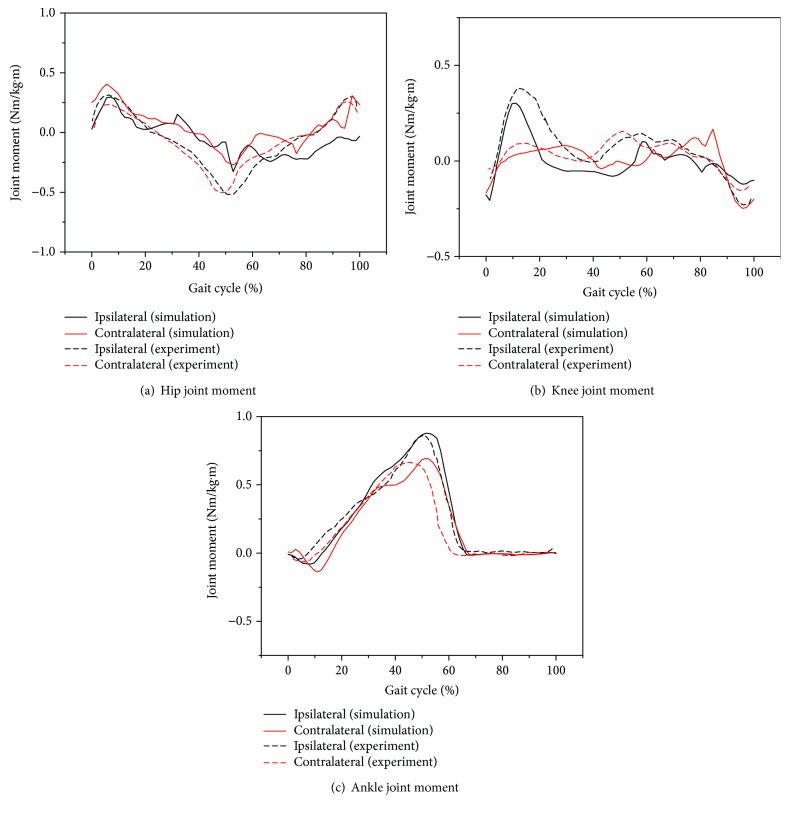
Comparison of joint moments between experimental tests and simulations.

Taking ground reaction forces and joint angles as input data, the inverse dynamic simulation was implemented with the model. The simulation results showed a good agreement with the experimental data regarding the joint moment as shown in Figure 3.  Table 2 shows the RMSE (root mean square error) values between the simulation results and the experimental data. The values are small except for the hip joint moment. The hip joint moment is also acceptable when considering the significant physical difference of the individuals.

A computed muscle control method was then used to calculate a set of desired muscle-tendon forces (*f*_des_) within the range of feasible forces [12], while the cost function is as follows:

(3) $$\begin{array}{c} J=\overset{m}{\underset{i=1}{\sum }}V_{i}{\left[a_{i}\left(t+T\right)\right]}^{2}, \end{array}$$

where *V*_*i*_ is the volume of muscle *i* and *a*_*i*_(*t* + *T*) is the activation of muscle *i* at *t* + *T* corresponding to the desired muscle force *f*_des_. These muscle forces were finally inputted into the finite element model as dynamic boundary conditions during the entire gait cycle simulation.

### 2.4. Finite Element Analysis with Gait Effects

**Figure 4:**
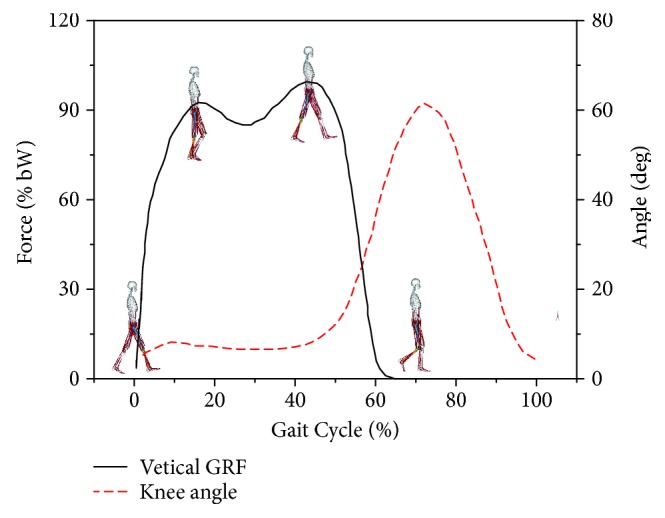
Four moments corresponding to typical GRFs and knee angles.

Regarding the ground reaction force and joint angles noted in the aforementioned experimental test, four typical gait states (0%, 18%, 45%, and 70% time steps of the gait cycle) were chosen to evaluate bone stress distribution during a gait cycle (Figure 4).

At 0% of the gait cycle, the human body gradually gets in contact with the ground and the flexion angle is at the minimum value during the gait cycle. At 18% of the gait cycle, the human body stands on a single leg and the first peak of the ground reaction force occurs. At 45% stage, the ground reaction force reaches its second peak. At 70% of the gait cycle, the maximum flexion angle of the knee joint during the gait appears. The related muscle forces, their insertion points, and executing directions were then extracted from these stages. The force levels of 28 muscles are listed in Table 3.

**Figure 5:**
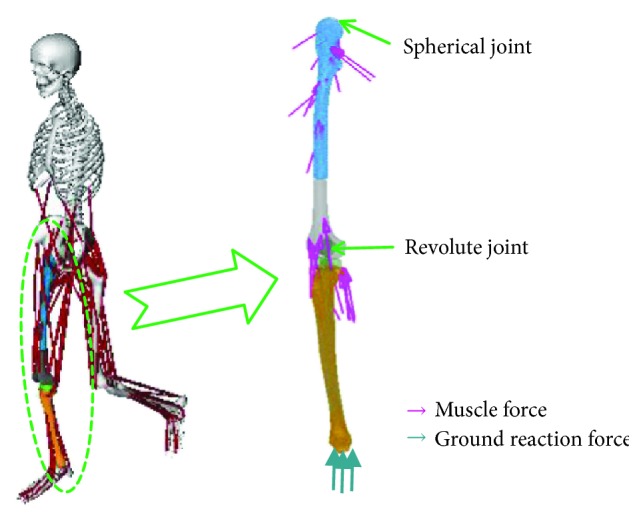
Definition of the finite element model with musculoskeletal analysis results.

The loading environment of the femur-prosthesis-tibia model is defined as shown in Figure 5. The ground reaction force was applied to the distal tibia. A spherical hinge with similar stiffness of the hip joint was created at the top of the femur to model the hip joint. Each muscle force was implemented on the insertion region with the direction along the action line.

## 3. Results and Discussions

**Figure 6:**
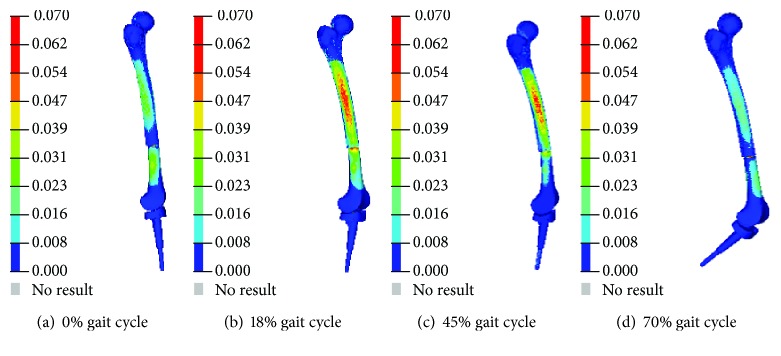
Stress distribution of the femur during the gait cycle.

**Figure 7:**
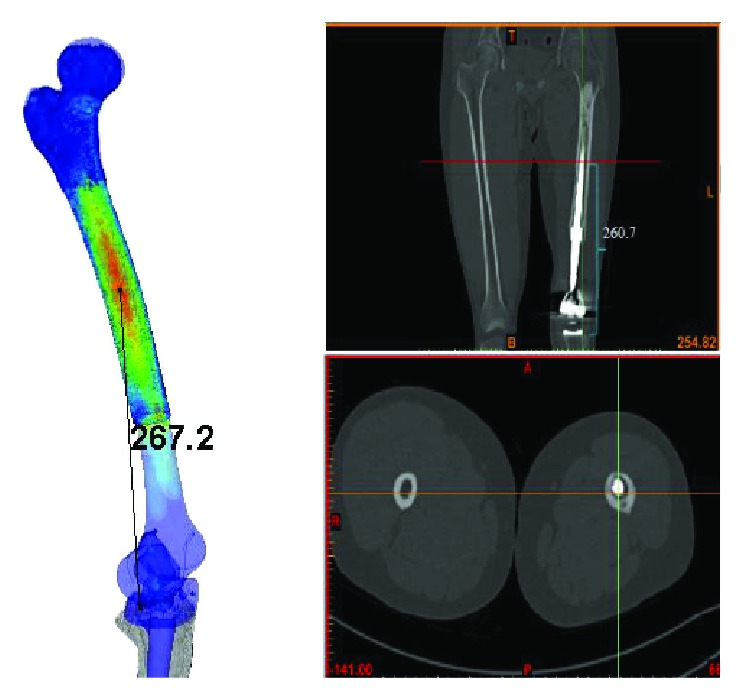
Comparison of the femur puncture injury with simulation results.

Concerning the four typical moments of the gait cycle, the maximum stress of each stage varied from 33.1 MPa to 70.6 MPa (Figure 6). These are extremely lower than the simulation results of maximum 102 MPa with simple fixed boundary conditions and a half body loading situation as reported by our previous study [13]. The maximum stress of 70.6 MPa during the whole gait cycle appeared at the 45% gait stage with the largest ground reaction force. At the 70% gait stage, the femur stress showed a minimum value of 33.1 MPa due to the swing of the lower limb without ground reaction force. The stress distribution of the femur in the four stages was similar. The concentration regions of the stress were all located at the femur shaft with a vertical distance around 267.2 mm to the tibial plateau (Figure 7). This indicated the high injury risk of this region. As shown in the CT scan results of the patient, the start position of the femoral penetration was around 260 mm which is in good agreement with the simulation results.

**Figure 8:**
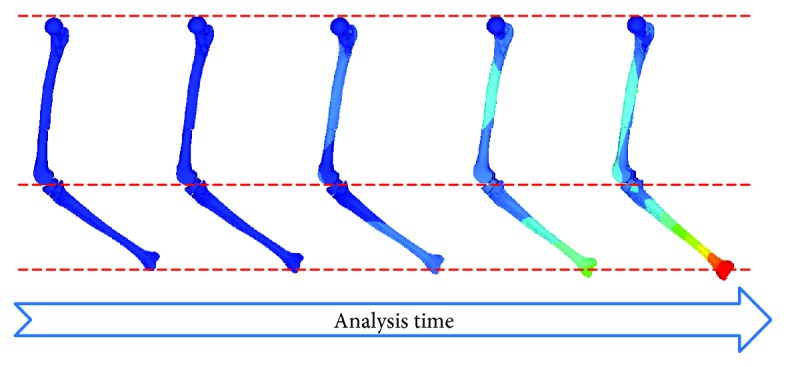
Dynamic movement of the femur-prosthesis-tibia model during gait stage analysis.

Recent studies performed several dynamic finite element analyses with relatively simple planar feet models to investigate stress and strain distributions of bones during the whole gait [14–17]. These models rotated around the defined joint center and were driven by joint forces and moments to simulate the gait movement. However, one of these studies pointed out that the accuracy of dynamic FE analysis was not always better than that of quasistatic FE analysis during the whole gait [17]. It indicated that the simplification of these models caused the deviations of dynamic simulation results. With the present method which is the coupled simulation, the femur-prosthesis-tibia complex can be considered in realistic loading environments with biofidelic muscle forces, ground reaction force, and hip joint constraint. In the example shown in Figure 8, the present loading conditions led to a slight movement of the complex during the simulations for all four gait stages. These slight movements coincided with the moving trail of the actual gait shown by musculoskeletal analysis. Moreover, the time when the stress concentration position did not significantly change was regarded as an important moment for recording the stress value in the present study. To access more accurate biomechanical responses, a complex finite element model which contains most muscles with validated activation levels is expected in the future study to analyze the stress distribution during the gait cycle. Thus, a continuous and holonomic gait analysis can be obtained through this model.

The stress concentration in the femur can be mainly due to the interaction between the femur and knee prosthesis. So the stress level of the femur would be related to the parameters of the knee prosthesis, including the curvature and length of the prosthetic stem. In order to predict the femur injury risk, it is meaningful to quantitatively predict the bone stress value with a specific implant model. According to the fatigue stress-life S/N data of human bones reported by Halloran et al. [18], the bone is expected to fail after about 10^7^ loading cycles with the fatigue stress around 70.61 MPa. Supposing that the patient walks 10 thousand steps a day and every step is regarded as one loading cycle, a femoral perforation might take place about three years later. This estimated time is close to the real perforation time of the patient. All these indicated that the present coupling simulation method is helpful to define realistic loading environments and estimate the injury risk of the patient with endoprosthetic knee replacement during walking and could also be extended to other implant analyses and structural optimizations in the future. In addition, on the basis of the geometric model and material density, we considered the influence of inertia and the center of gravity when introducing the prosthesis model in the musculoskeletal model for the gait analysis. But for the part of cutting the muscles and bones, it is difficult to estimate; hence, their influences were not included in the present study. The detailed modeling on this anatomy change can be further investigated in the future.

## 4. Conclusions

This study investigated femur penetration injury of the patient after endoprosthetic knee replacement by combining musculoskeletal dynamics and structural finite element analysis. A subject-specific finite element model of the femur-prosthesis-tibia complex was first established by medical image data. Then, reverse dynamic gait analysis for the patient wearing knee endoprosthesis was performed with the adjusted musculoskeletal model to define physiological realistic loading environments for the femur-prosthesis-tibia complex. Finally, the maximum stress was found to vary from 33.14 MPa to 70.61 MPa through the finite element analysis of four typical gait stages. The high injury risk regions with maximum stress in the simulations were similar to the patient injury data. All these indicated that the present method is valuable to determine structural design of joint replacement and surgery strategy.

## Figures and Tables

**Table 1:** Material parameters of the bones, prosthesis, and bone cement.

Models		*ρ* (kg·m^−3^)	Poisson's ratio	*E* (GPa)
Femur	Cancellous bone	214.5	0.30	0.127
	Cortical bone	321.4	0.36	1.904
	Neck of the femur	621.3	0.32	3.681
	Shaft of the femur	921.2~1521	0.36	5.458~9.012
Tibia	Cancellous bone	100	0.30	0.49
	Cortical bone	390	0.36	2.3
	Shaft of the tibia	700~1600	0.36	4.2~9.6
Prosthesis	Broach	4.51 ∗ 10^3^	0.32	113
	Tibial plateau gasket	960	0.3	0.5
	Tibial plateau	8.8 ∗ 10^3^	0.32	70
Bone cement		1190	0.28	0.3

**Table 2:** RMSE values between the simulation and experimental data.

	Ipsilateral	Contralateral
RMSE of hip joint moment (%)	18.5	15.5
RMSE of knee joint moment (%)	10.9	7.8
RMSE of ankle joint moment (%)	5.7	10.7

**Table 3:** Muscle forces of four typical times.

Muscle name	Force of different times in gait (*N*)			
	0%	18%	45%	70%
Gluteus maximus-1	27.11	11.36	13.25	31.44
Gluteus maximus-2	41.66	15.21	18.39	49.08
Gluteus maximus-3	25.63	8.38	15.11	38.61
Gluteus medius-1	22.73	36.67	17.35	62.65
Gluteus medius-2	33.46	20.67	22.52	45.71
Gluteus medius-3	53.39	22.65	36.19	49.41
Gluteus minimus-1	9.18	18.77	10.19	20.42
Gluteus minimus-2	13.62	19.09	11.80	22.54
Gluteus minimus-3	21.76	14.43	13.29	26.08
Musculi adductor longus	18.30	36.34	41.57	11.01
Musculi adductor brevis	15.62	24.14	28.17	13.91
Musculi adductor magnus-1	11.28	9.45	20.16	12.92
Musculi adductor magnus-2	10.42	7.33	14.74	18.65
Musculi adductor magnus-3	28.26	11.27	15.80	32.79
Pectineus	8.06	14.51	16.15	4.84
Anterior superior spine	49.85	91.16	119.65	17.82
Iliopsoas	51.15	88.26	94.38	18.51
Quadratus femoris	28.69	8.64	29.98	7.96
Rectus femoris	51.87	85.14	89.97	42.21
Piriformis	35.50	10.15	31.68	31.46
The long head of the biceps femoris	58.27	18.77	21.06	64.94
The short head of the biceps femoris	34.52	37.74	30.35	48.37
Sartorius	7.09	9.52	7.99	6.60
Musculi tensor fasciae latae	8.09	24.52	33.04	9.83
Gracilis	7.94	7.99	8.59	9.96
Semitendinosus	30.59	12.88	13.99	32.31
Semimembranosus	72.99	20.73	18.08	60.48
Obturator internus	13.42	8.84	16.02	6.62

## Data Availability

The data used to support the findings of this study are available from the corresponding author upon request.
